# Personality and breast cancer screening in women of the GAZEL cohort study

**DOI:** 10.1002/cam4.1268

**Published:** 2017-12-26

**Authors:** Cédric Lemogne, Monica Turinici, Henri Panjo, Charlotte Ngo, Florence Canoui‐Poitrine, Jean‐Christophe Chauvet‐Gelinier, Frédéric Limosin, Silla M. Consoli, Marcel Goldberg, Marie Zins, Virginie Ringa

**Affiliations:** ^1^ Faculté de Médecine Sorbonne Paris Cité Université Paris Descartes Paris France; ^2^ Psychiatry department Paris‐Ouest University Hospitals AP‐HP Paris France; ^3^ Inserm, U894 Paris France; ^4^ UVSQ CESP, Inserm, INED Université Paris‐Saclay Univ. Paris‐Sud le Kremlin‐Bicêtre France; ^5^ Gynecologic Surgery Department Paris‐Ouest University Hospitals AP‐HP Paris France; ^6^ Public Health department Henri‐Mondor Hospital AP‐HP Créteil France; ^7^ UPEC, DHU A‐TVB, IMRB, EA7376 CEpiA (Clinical Epidemiology And Ageing) Paris‐Est University F‐94000 Créteil France; ^8^ Psychiatry unit Neurosciences department, Marion building CHU Le Bocage Dijon France; ^9^ Laboratory of psychopathology and medical psychology (IFR 100) Bourgogne University Dijon France; ^10^ Inserm, UMS 011 Population‐based Epidemiological Cohorts Villejuif France; ^11^ Inserm, UMR 1168, VIMA Villejuif France

**Keywords:** Breast neoplasms, cancer, cohort studies, early detection of cancer, mammography, oncology, personality

## Abstract

The potential benefit of breast cancer screening is mitigated by the risk of false positives and overdiagnosis, thus advocating for a more personalized approach, based on the individual benefit‐harm balance. Since personality might influence the women's appraisal of this balance, this prospective observational cohort study examined whether it could influence mammography use. A total of 2691 postmenopausal women of the GAZEL Cohort Study completed the Bortner Type A Rating Scale and the Buss and Durkee Hostility Inventory in 1993. Associations between personality scores and subsequent mammography use, self‐reported through up to five triennial follow‐up questionnaires, were estimated with Odds Ratio (OR) and 95% confidence interval (CI) with logistic mixed model regressions, adjusting for age, occupational grade, marital status, family history of breast cancer, age at menarche, age at first delivery, gynecological follow‐up, hormone therapy use, and depressive symptoms. Individual propensity scores were used to weight the analyses to control for potential selection biases. More than 90% of the participants completed at least two follow‐up questionnaires. Type A personality, but not hostility, was associated with mammography use in both univariate (crude OR [95% CI]: 1.62 [1.24–2.11], *P *< 0.001) and multivariate analyses (OR [95% CI]: 1.46 [1.13–1.90], *P *< 0.01). Type A personality traits (i.e., sense of time urgency, high job involvement, competitiveness) independently predicted mammography use among postmenopausal women. While paying more attention to the adherence of women with low levels of these traits, clinicians may help those with higher levels to better consider the risks of false positives and overdiagnosis.

## Introduction

Breast cancer is one of the most frequent cancer worldwide and the first cause of death by cancer among women in France 1. As a consequence, breast cancer screening based on repeated mammography has been extensively recommended over the two last decades, especially among women aged 50–74 2, 3. French national breast cancer screening program has been implemented since 1994 and gradually spread throughout the country from 1994 to 2004. This national screening program thus covers all parts of France since 2004. It only concerns women without risk factors and without symptoms. All women aged 50–74 receive a mailed invitation from local management structures to go on a mammogram, along with the list of approved radiologists in the surrounding area. The screening includes a clinical examination and a mammogram (X‐ray from the side and top of each breast), which is analyzed while the woman is still at the clinic for her mammography appointment. If this first analysis detects an abnormality, the result is immediately given to the patient while she is still at the clinic and the necessary additional examinations are proposed. If this first analysis is normal, a second analysis is subsequently performed by another radiologist. If the second analysis detects an abnormality, the patient is convoked for additional examinations. If mammograms are normal after a double reading, the woman is invited again after 2 years. This screening is free of charge and if it gives rise to further examinations, they are reimbursed.

Although such programs were found to reduce mortality of 20% in randomized controlled trials 3, this benefit might be lower in real‐life setting and potentially outweighed by the psychological and physical consequences of false positives and overdiagnosis (i.e., cancers detected at screening that would not have otherwise become clinically relevant in the woman's lifetime) 4. As a consequence, some experts advocate for a more personalized management of breast cancer screening, including information on the expected benefit‐harm balance at an individual level 3, 4, 5, 6, 7. To achieve this goal, it is therefore critical to better understand the factors that influence the appraisal of this balance.

Despite physicians’ recommendations, a substantial proportion of eligible women do not perform repeated mammography, whereas some noneligible women (e.g., aged less than 50 or more than 74) do. Studies that examined the psychosocial factors that may influence these behaviors found lower adherence to repeated mammography among women of low socioeconomic status, who are not living in couple, belong to an ethnic minority and have a reduced access to health care 8, 9. Regarding more psychological factors, adherence is predicted by perceived breast cancer risk, which is increased by aging, a family history of breast cancer, education and breast cancer worry 10. Although worry is associated with a small, yet significantly greater likelihood of screening 11, there is still some debate about whether it could promote or impede cancer screening 12, 13. Other psychological factors that may hamper adherence to repeated mammography include high perceived stress and low sense of control 8, 9, 14. Surprisingly, however, little is known about the role of personality, defined as a stable person's characteristic pattern of behavior, thoughts, and feelings.

This study took advantage of the French GAZEL cohort 15 to examine in a prospective way whether mammography use would be influenced by Type A personality and various facets of hostility. These personality constructs were measured in GAZEL participants in 1993. Thereafter, most of GAZEL women have been followed for several years via repeated self‐administered questionnaires with specific questions regarding adherence to breast cancer screening through mammography. The Type A personality is defined by the combination of several traits encompassing a sense of time urgency, high job involvement, hard driving, need for achievement, ambition, and competitiveness. In the context of health behaviors, Type A is associated with problem‐focused coping strategies 16 and was found to predict hormone therapy use among postmenopausal women 17. It is also associated with conscientiousness 18, a personality dimension characterized by control, organization, and assiduousness. Therefore, Type A personality was expected to be associated with a greater likelihood to perform repeated mammography, as an active way to deal with the risk of breast cancer encouraged by physicians’ recommendations. Inversely, based on evidence suggesting that hostility is associated with poor therapeutic alliance 19 and poor medical adherence 20, hostility was expected to be associated with a lower likelihood to adhere to breast cancer screening through repeated mammography.

## Methods

### Population

Details of the GAZEL cohort study are available elsewhere 21. Briefly, the target population consisted of 44,992 employees of the French national gas and electricity company (31,411 men aged 40–50 and 13,511 women aged 35–50). The study protocol was approved by the French authority for data confidentiality (“Commission Nationale Informatique et Liberté”) and by the Ethics Evaluation Committee of the “Institut National de la Santé et de la Recherche Médicale” (INSERM) (IRB0000388, FWA00005831). At inclusion in 1989, 20,625 employees (45.8%) (15,011 men and 5614 women) gave written informed consent to participate in the GAZEL cohort study. In 1990, a prospective survey entitled “Women and Their Health” began within this cohort. All female participants who were at least 45 years old were initially included; all GAZEL women who reached the age of 45 between 1990 and 1996 were subsequently included. Data from up to seven mailed questionnaires – at inclusion (in 1990) and every 3 years (up to 2008) – were used to determine adherence to breast cancer screening through mammography. At each follow‐up questionnaire, participants gave a yes/no answer to the following question: “Over the past three years, did you have at least one mammography?” In 1993, questionnaires were mailed to the 20,488 living members of the GAZEL cohort to assess Type A personality and hostility 22. Women of the GAZEL cohort were included in this study if they had completed the assessment of both Type A personality and hostility in 1993 and completed at least one follow‐up questionnaire of the “Women and Their Health” survey with no missing data regarding time‐dependent variables (see below) before any personal history of breast cancer. In this study, only follow‐up questionnaires subsequently completed after the personality assessment in 1993 were considered (i.e., up to five questionnaires from 1996 to 2008). Follow‐up questionnaires obtained after a diagnosis of breast cancer were censored.

### Psychological variables

The Type A personality was assessed with the Bortner Type A Rating Scale (BTARS) 23. It consists of 14 items each comprising two statements with a graded scale between the two statements (24‐point scale in the original version, 6‐point scale in the version adapted for the GAZEL cohort). Examples of statements include “never late” versus “casual about appointments.” Importantly, the BTARS captures time urgency, job involvement, hard driving, need for achievement, ambition, and competitiveness, but not hostility. The sum of the 14 items yields a global score ranging from 14 to 84. This scale was translated and validated for the French population against the Friedman and Rosenman structured interview for assessing Type A, agreement observed 71.5% 24. Higher scores indicate higher levels of Type A personality traits.

Hostility was assessed with the Buss and Durkee Hostility Inventory (BDHI). The BDHI was previously validated in French in 408 randomly selected participants of the GAZEL cohort study 22. The BDHI is composed of 75 items with “true‐false” answers 25. It has eight subscales, seven of which are designed to measure different components of hostility: assault, verbal aggression, indirect hostility, irritability, negativism, resentment, and suspicion. Higher scores indicate higher hostility. The sum of these seven subscales leads to a “total hostility” score with a high 3‐month test‐retest reliability (*r* = 0.87) 22. Several factor analyses identified two overarching factors, namely “behavioral” (i.e., hostile behaviors) and “cognitive” hostility (i.e. hostile thoughts), formed by the first three subscales (i.e., assault, verbal aggression, indirect hostility) and the last two subscales (i.e., resentment, suspicion), respectively 26. In the GAZEL cohort study, the internal consistency was high for total, behavioral and cognitive hostility scores (*α *= 0.87, 0.78, and 0.77, respectively) 27.

Depressive symptoms at the time of personality assessment may bias this assessment and confound the association between personality and health outcomes 28. For instance, in the context of breast cancer, depression has been associated with a delayed diagnostic 29 and poor adherence to adjuvant endocrine therapy 30. Therefore, depressive symptoms in 1993 were also considered as a covariate. Depressive symptoms were assessed with the French version of the 20‐item Center of Epidemiologic Studies Depression Scale (CESD), which has been designed for use in community studies with a high internal consistency ranging from *α *= 0.8 to *α *= 0.9 across samples and a moderate 2‐week test‐retest reliability (*r* = 0.51) 31, 32. The CESD asks participants how often they have experienced specific symptoms during the previous week (e.g., “I felt depressed,” “I felt everything I did was an effort,” “My sleep was restless”). Responses range from 0 (“hardly ever”) to 3 (“most of the time”), yielding a global score ranging from 0 to 60 with higher scores indicating higher level of depressive symptoms.

### Other covariates

Age, occupational grade (blue‐collar and clerical staff, first‐line supervisors and sales representatives, or management and training), and marital status (living as couple or not) were obtained from company human resources records. Occupational grade is a useful proxy for socioeconomic status as it integrates the educational achievements, the skills required to obtain a job, the long‐term associated rewards (including, but not limited to, income), and several job characteristics, such as working conditions and decision‐making latitude 33. Other variables were self‐reported: family history of breast cancer (mother, sister, maternal grandmother, mother's sister: yes or no), age at menarche (<11 or ≥11 years old), age at first delivery (nulliparous, <20, 20–29, or ≥30 years old), gynecological follow‐up (no, yes but not by a gynecologist, yes by a gynecologist), and hormone therapy use for menopause.

### Statistical methods

All analyses were performed with R Foundation for Statistical Computing, Vienna, Austria. All tests were two‐sided with *α* = 0.05. The association between independent variables and mammography over the past 3 years at each available follow‐up (yes vs. no) was estimated with Odds Ratio (OR) and 95% confidence interval (CI) computed through logistic mixed model regressions to take into account the correlation between measurements within the same woman. Age, occupational grade, marital status, specialized gynecological follow‐up, and hormone therapy use were considered as time‐dependent variables, whereas psychological variables (i.e., personality and depressive symptoms), family history of breast cancer, age at menarche, and age at first delivery were considered as stable throughout the follow‐up. Age and psychological variables, including Type A personality and hostility, were analyzed as continuous variables and rescaled using the difference between the 25th and the 75th percentile as the unit, in order to yield meaningful OR 33. The other variables were analyzed as nominal variables. Since completion of follow‐up questionnaires may be associated with screening adherence as well, all the analyses were weighted in order to control for potential selection biases. Individual propensity score were computed with a multivariate ordinary logistic regression predicting the completion of all follow‐up questionnaires (vs. missing at least one questionnaire). Then, each subject was given a weight equal to the multiplicative inverse of the propensity score 34. Model 1 was adjusted for sociodemographic variables (i.e., age, occupational grade, marital status). Models 2, 3, and 4 added nonmodifiable breast cancer risk factors, gynecological follow‐up, and hormone therapy use, respectively, to model 1. Breast cancer risk factors were included in the analysis as they might have influenced women's motivation to perform a mammography. Model 5 simultaneously included all these variables. Finally, model 6 further included depressive symptoms.

## Results

Among the 5559 women of the GAZEL cohort still alive in 1993, a total of 3399 completed the personality questionnaires, including 2691 women who subsequently completed at least one follow‐up questionnaire with no missing data regarding time‐dependent variables before any personal history of breast cancer and thus participated in this study. The characteristics of the participants are displayed in Table 1. More than 90% of the participants had completed at least two follow‐up questionnaires and a total of 8188 follow‐up questionnaires were collected. Mammography use over the past 3 years was reported in 7759 cases (94.8%) with a mean number of mammography of 2.9 per woman over the whole follow‐up period.

**Table 1:** Characteristics of the participants at inclusion in this study (*N* = 2691) from the GAZEL cohort

Continuous variables	Mean	SD
Age	51.5	2.8
Type A personality	54.3	7.4
Total hostility	29.6	9.5
Behavioral hostility	6.8	3.4
Cognitive hostility	14.0	5.2
Depressive symptoms	16.4	10.5

Discrete variables	*N*	%
Occupational grade		
Blue‐collar workers, clerks	609	22.6
First‐line supervisors, sales representatives	1839	68.3
Management or training	243	9.0
Marital status		
Single, separated, divorced, widowed	661	24.6
Living as couple	2030	75.4
Age at menarche		
<11 years old	97	3.6
≥11 years old	2594	96.4
Age at first delivery		
Nulliparous	295	11.0
<20 years old	125	4.6
20–29	1978	73.5
≥30 years old	293	10.9
Family history of breast cancer		
No	2275	84.5
Yes	416	15.5
Gynecological follow‐up		
None	177	6.6
By a gynecologist	2300	85.5
By a nongynecologist	214	7.9
Hormone therapy use		
No	848	31.5
Yes	1843	68.5
Number of completed follow‐up questionnaires		
1	242	9.0
2	768	28.5
3	552	20.5
4	891	33.1
5	238	8.8

Type A score was the only personality variable associated with mammography use in univariate analyses (OR [95% CI]: 1.62 [1.24–2.11], *P *< 0.001), as were age, marital status, gynecological follow‐up, hormone therapy use, and depressive symptoms (Table 2). An analysis based on quartiles suggested that the association between Type A score and mammography use was consistent with a linear relationship with OR [95% CI] increasing from the first quartile (reference) to the second (1.16 [0.67–2.00]), third (2.32 [1.32–4.07]), and fourth quartile (2.50 [1.41–4.40]) (*P* for linear trend < 0.001 with OR [95% CI]: 1.41 [1.18–1.69] for a one‐quartile increment). There was no significant interaction between Type A personality and the other variables. In contrast, there was no crude association between mammography use and cognitive (OR [95% CI]: 1.00 [0.94–1.06]), behavioral (1.03 [0.99–1.07]), or total hostility (1.02 [0.98–1.04], all *P *> 0.05). These associations were not significant in the model 1 either (all *P *> 0.05), thus preventing examination of more complex models for hostility measures.

**Table 2:** Associations between mammography and each variable in this study (from the GAZEL cohort)

	Crude associations		Model 1		Model 2		Model 3		Model 4		Model 5		Model 6	
	OR	95% CI	OR	95% CI	OR	95% CI	OR	95% CI	OR	95% CI	OR	95% CI	OR	95% CI
Type A personality^a^	1.62***	1.24–2.11	1.70***	1.30–2.26	1.64***	1.23–2.20	1.44**	1.10–1.90	1.67***	1.26–2.20	1.50**	1.16–2.00	1.46**	1.13–1.90
Age^a^	1.90***	1.66–2.15	1.92***	1.70–2.20	1.90***	1.70–2.16	2.60***	2.21–3.00	1.91***	1.68–2.20	2.43***	2.09–2.82	2.64***	2.26–3.08
Occupational grade														
Blue‐collar workers, clerks	0.65	0.40–1.04	0.70	0.41–1.10	0.62	0.40–1.02	0.80	0.50–1.24	0.66	0.40–1.08	0.86	0.55–1.36	0.74	0.47–1.17
First‐line supervisors, sales representatives	Ref.		Ref.		Ref.		Ref.				Ref.		Ref.	
Management	0.67	0.34–1.35	0.61	0.30–1.30	0.55	0.30–1.15	0.60	0.30–1.17	0.61	0.30–1.24	0.60.	0.30–1.20	0.60	0.30–1.16
Marital status														
Living as couple	Ref.		Ref.		Ref.		Ref.				Ref.		Ref.	
Single, separated, divorced, widowed	0.61*	0.41–0.88	0.60**	0.40–0.83	0.56**	0.40–0.84	0.68	0.46–1.01	0.57**	0.40–0.85	0.78**	0.53–1.15	0.70	0.50–1.04
Age at menarche														
<11 years old	1.13	0.38–3.31			0.86	0.30–2.65					0.83	0.30–2.22	0.88	0.32–2.43
≥11 years old	Ref.				Ref.						Ref.		Ref.	
Age at first delivery														
Nulliparous	0.78	0.42–1.47			0.83	0.42–1.61					1.36	0.73–2.54	1.14	0.62–2.10
<20 years old	1.64	0.61–4.41			1.65	0.60–4.70					2.03	0.74–5.60	1.90	0.70–5.10
20–29	Ref.				Ref.						Ref.		Ref.	
≥30 years old	1.86	0.94–3.70			1.90	0.90–3.95					1.95	0.97–3.88	1.51	0.77–2.94
Family history of breast cancer														
Yes	1.73	1.00–3.06			1.76	1.00–3.20					1.88*	1.06–3.32	1.67	0.96–2.92
No	Ref.				Ref.						Ref.		Ref.	
Gynecological follow‐up														
None	0.03***	0.02–0.04					0.01***	0.01–0.02			0.02***	0.01–0.03	0.02***	0.01–0.03
By a gynecologist	Ref.						Ref.				Ref.		Ref.	
By a nongynecologist	0.26***	0.17–0.40					0.20***	0.10–0.30			0.20***	0.11–0.30	0.20***	0.13–0.30
Hormone therapy use														
Yes	3.25***	2.14–5.00							2.73***	1.76–4.24	2.00**	1.30–2.90	1.90**	1.30–2.83
No	Ref.								Ref.		Ref.		Ref.	
Depressive symptoms^a^	0.77*	0.63–0.94											0.91	0.73–1.12

a OR value are given normalized according to the interval between the 25th and the 75th percentile; *P < 0.05; **P < 0.01; ***P < 0.001.

Table 2 also displays multi‐adjusted models. Adjusting for age, occupational grade, and marital status (model 1), mammography remained significantly and positively associated with higher Type A scores (OR [95% CI]: 1.70 [1.30–2.26], *P *< 0.001). Further adjustment for nonmodifiable breast cancer risk factors, gynecological follow‐up, hormone therapy use, and depressive symptoms yielded similar results, with ORs ranging from 1.44 to 1.67 for the association of Type A personality with mammography use (Table 2). Indeed this association remained significant when all the variables were entered into model 6 (OR [95% CI]: 1.46 [1.13–1.90], *P *< 0.01).

In sensitivity analyses, the association of Type A personality with mammography use remained significant when further adjusting model 5 for total hostility (OR [95% CI]: 1.45 [1.10–1.92], *P *< 0.01), cognitive hostility (1.38 [1.05–1.80], *P *< 0.05) or behavioral hostility (1.32 [1.04–1.80], *P *< 0.05).

## Conclusions

This study aimed to examine whether mammography use would be influenced by Type A personality and various facets of hostility in a large prospective cohort of women. We hypothesized that mammography use would be positively and negatively associated with Type A personality and hostility, respectively. On the basis of logistic mixed model regressions accounting for within‐subject autocorrelation, we found strong support for the former hypothesis. In multivariate analyses adjusting for potential confounders including age, occupational grade, marital status, nonmodifiable breast cancer risk factors, gynecological follow‐up, hormone therapy use, and depressive symptoms, Type A personality was significantly associated with an increase of 46% of the odds of mammography use. In addition, this association was independent from hostility measures.

One might have speculated about the role of several covariates, such as occupational grade or gynecological follow‐up, in confounding or mediating the association between Type A and mammography use. However, the slight OR reduction from the crude association to the multiadjusted OR in models 1–6 suggests that these covariates were unlikely to substantially account for this association. For instance, although Type A personality was not directly linked with an increased risk of breast cancer 35, it was found to predict hormone therapy use among postmenopausal women of the GAZEL cohort 17. The associated increase in breast cancer risk could thus have promoted greater adherence to cancer screening procedures. However, although hormone therapy use was associated with mammography use in this study, further adjustment for the former did not reduce the association of Type A personality with the latter. Our results rather suggest an independent and specific role of Type A per se. On the basis of studies that examined the behavioral, cognitive, and social correlates of Type A personality, several hypotheses might explain why women scoring high on these traits behave differently when it comes to adhere to breast cancer screening.

First, Type A personality is associated with greater levels of conscientiousness 18, a personality dimension that increases adherence to medical recommendation 36. Second, Type A personality may influence the appraisal of the benefit‐harm balance of repeated mammography. In the context of complex decision‐making involving several dimensions, Type A personality is associated with a tendency to prioritize one dimension over the others, then choosing the option with the highest value on this dimension 37. Breast cancer and its consequences may be more threatening for women with Type A personality traits, which are associated with job commitment, need for achievement and dominance 38. In addition, Type A personality is associated with an inclination toward problem‐focused coping strategies 16. Therefore, women with Type A personality may have been more prone to use mammography as an efficient way to prevent breast cancer to interfere with their lifestyle and goals. Interestingly, although adjustment for sociodemographic variables tended to increase the OR of the association of Type A personality with mammography use (i.e., from crude association to model 1), only adjustment for gynecological follow‐up (i.e., from crude association to model 3) resulted in an OR decrease. This is consistent with a partial mediation by gynecological follow‐up. Although this study was not designed to test this mediation hypothesis, this result is in line with the more global hypothesis that Type A personality is associated with more favorable health behaviors as long as active coping is adapted. Finally, there is experimental evidence that Type A individuals prefer to interact with other Type A individuals 39. Type A women may thus have been more likely to choose Type A physicians, thus promoting a congruent appraisal of the benefit‐harm balance of repeated mammography.

### Study limitations

First, psychological variables were measured through questionnaires rather than standardized interviews. More specifically, self‐report measures of Type A assess only parts of the overall behavior pattern, and this varies from one instrument to another 40. The BTARS captures time urgency, job involvement, and competitiveness dimensions, but not hostility. Second, although the GAZEL cohort covers all regions of France, various neighborhoods from small villages to large cities and a wide range of socioeconomic status and occupations, it is not representative of the general population as it includes only middle‐aged working individuals with employment security and excluded certain categories of the population (e.g., agricultural workers, self‐employed, foreigners). Such selection biases may explain for instance why mammography use was somewhat more frequent among GAZEL women than in the general population but are unlikely to account for its association with Type A personality. Third, several potential mediators of the association between Type A and hormone therapy use were not measured, such as sense of control, previous symptoms of problems with breasts, breast cancer worry, and awareness regarding breast cancer, or therapeutic alliance. Most of these factors, however, are more likely to be mediators than confounders of the association. Technically, a reduction in the association between personality and mammography use once adjusted for these variables would therefore suggest potential causal pathways linking personality to mammography use. Body mass index was not included because of missing data. In addition, mammography use was self‐reported and information biases cannot be formally excluded. Even if questionnaires completed after a diagnosis of cancer were discarded, we could also not formally differentiate between mammography screening and diagnostic mammography initiated after signs of breast cancer. Fourth, the generalization of our results outside of France may be questioned. For instance, 52% of the target population attended the French national program of breast cancer screening in 2010, whereas 10% underwent individual screening in a primary care setting. However, this program based on biennial mammograms for women aged 50–74 years is in line with recommendations in other high‐income countries. For instance, the US Preventive Services Task Force recommends biennial mammograms in women aged 50–74 2, whereas UK breast screening programs recommends triennial mammograms in women aged 50–70 years 3. Fifth, the data analyzed in this study were collected from 1996 to 2008 and may not be representative of the current situation in France. However, the French national program of breast cancer screening has not changed much since 2004 so that it likely that the conclusions of this study still hold.

### Clinical implications

Although the personality of the patient may not be critical for physicians when assessing the individual benefit‐harm balance of a mammography, it may substantially affect medical adherence afterwards. Our results suggest that Type A personality may be associated with mammography use among middle‐aged women, regardless of other predictors, including several breast cancer risk factors and gynecological follow‐up. Clinicians should be aware of the influence of the patient's personality in the decision‐making process regarding repeated mammography use. Type A personality, which is overtly characterized by a sense of time urgency, high job involvement, need for achievement, and competitiveness, can easily be recognized by physicians. While paying more attention to the risk of poor adherence of women with low levels of Type A personality traits, they may help women with higher levels of these traits to better consider the risks of both false positives and overdiagnosis, especially if they are aged less than 50 or more than 74. Having a false‐positive screening mammogram can cause long‐lasting psychological distress and result in a decreased likelihood to perform subsequent routine assessment 6. From a research perspective, the fact that some psychological variables may promote mammography use should be remembered when interpreting the results of epidemiological studies linking these variables with cancer incidence 35, 41. Future studies may examine whether personality might also predict adherence to other cancer screening programs, including controverted ones such as prostate cancer screening.

## Conflict of Interest

The authors have no conflict of interests to disclose.
